# The Volume of Hippocampal Subfields in Relation to Decline of Memory Recall Across the Adult Lifespan

**DOI:** 10.3389/fnagi.2018.00320

**Published:** 2018-10-10

**Authors:** Fenglian Zheng, Dong Cui, Li Zhang, Shitong Zhang, Yue Zhao, Xiaojing Liu, Chunhua Liu, Zhengmei Li, Dongsheng Zhang, Liting Shi, Zhipeng Liu, Kun Hou, Wen Lu, Tao Yin, Jianfeng Qiu

**Affiliations:** ^1^Medical Engineering and Technology Research Center, Taishan Medical University, Taian, China; ^2^Imaging-X Joint Laboratory, Taian, China; ^3^College of Radiology, Taishan Medical University, Taian, China; ^4^Institute of Biomedical Engineering, Chinese Academy of Medical Sciences and Peking Union Medical College, Tianjin, China; ^5^College of Mechanical and Electronic Engineering, Shandong University of Science and Technology, Qingdao, China; ^6^School of Basic Medical Sciences, Taishan Medical University, Taian, China

**Keywords:** hippocampal subfields, lifespan, immediate recall, delayed recall, memory decline

## Abstract

**Background:** The hippocampus is an important limbic structure closely related to memory function. However, few studies have focused on the association between hippocampal subfields and age-related memory decline. We investigated the volume alterations of hippocampal subfields at different ages and assessed the correlations with Immediate and Delayed recall abilities.

**Materials and Methods:** A total of 275 participants aged 20–89 years were classified into 4 groups: Young, 20–35 years; Middle-early, 36–50 years; Middle-late, 51–65 years; Old, 66–89 years. All data were acquired from the Dallas Lifespan Brain Study (DLBS). The volumes of hippocampal subfields were obtained using Freesurfer software. Analysis of covariance (ANCOVA) was performed to analyze alterations of subfield volumes among the 4 groups, and multiple comparisons between groups were performed using the Bonferroni method. Spearman correlation with false discovery rate correction was used to investigate the relationship between memory recall scores and hippocampal subfield volumes.

**Results:** Apart from no significant difference in the left parasubiculum (*P* = 0.269) and a slight difference in the right parasubiculum (*P* = 0.022), the volumes of other hippocampal subfields were significantly different across the adult lifespan (*P* < 0.001). The hippocampal fissure volume was increased in the Old group, while volumes for other subfields decreased. In addition, Immediate recall scores were associated with volumes of the bilateral molecular layer, granule cell layer of the dentate gyrus (GC-DG), cornus ammonis (CA) 1, CA2/3, CA4, left fimbria and hippocampal amygdala transition area (HATA), and right fissure (*P* < 0.05). Delayed recall scores were associated with the bilateral molecular layer, GC-DG, CA2/3 and CA4; left tail, presubiculum, CA1, subiculum, fimbria and HATA (*P* < 0.05).

**Conclusion:** The parasubiculum volume was not significantly different across the adult lifespan, while atrophy in dementia patients in some studies. Based on these findings, we speculate that volume changes in this region might be considered as a biomarker for dementia disorders. Additionally, several hippocampal subfield volumes were significantly associated with memory scores, further highlighting the key role of the hippocampus in age-related memory decline. These regions could be used to assess the risk of memory decline across the adult lifespan.

## Introduction

The hippocampus is an important limbic structure (Richter-Levin, 2004) with a critical role in memory and is particularly vulnerable to aging (Eichenbaum, 2004; Mueller et al., 2011; Malykhin et al., 2017; Zammit et al., 2017). It is regarded as an amalgamated structure (Van, 2004), but few studies have focused on volume changes in hippocampal subfields with normal aging and their relationships with age-related memory decline.

The mammalian hippocampus consists of several subfields with different memory functions (Hunsaker et al., 2008; Yassa and Stark, 2011; Engvig et al., 2012; Reagh et al., 2014). For example, Hunsaker et al. (2008) found that CA1 and CA3 both contribute to episodic memory processing by training memory ability in rats. Another study reported that rats with dentate gyrus (DG) lesions were unable to distinguish between training and testing environments (Yassa and Stark, 2011).

Numerous human studies have reported the functions of hippocampal subfields in various disorders. For example, histological studies suggest that AD variably affects different hippocampal subfields. Especially in the early stage, tangle accumulation and neuron loss are more prominent in the CA1 and subiculum (Rössler et al., 2002; Schönheit et al., 2004). Mueller et al. (2012) suggested that auditory Immediate recall was associated with the CA3 and DG, while auditory Delayed recall and auditory Delayed recognition were related to the CA1 in subjects with temporal lobe epilepsy with hippocampal sclerosis. In those without hippocampal sclerosis, to a lesser degree, auditory Immediate recall was associated with the CA3 and DG, whereas auditory Delayed recall and recognition were more closely related to the fusiform gyrus. In a recent study, the CA1 was implicated in Immediate and Delayed recall of verbal memory in patients with left hippocampal sclerosis, while for patients with right hippocampal sclerosis, the CA1 and epilepsy duration were related to visual memory (Comper et al., 2017). Carlesimo et al. (2015) found no significant correlation between hippocampal subfield volumes and Immediate and Delayed recall scores in healthy participants. They reported that AD patients had significant correlations with both types of memory performance and the CA2-3, CA4-DG, and subiculum. The presubiculum was only associated with Delayed recall. Finally, they found that patients with mild cognitive impairment showed significant correlations between Immediate recall and presubiculum and subiculum volumes. These preliminary studies suggest that a detailed volumetric study of hippocampal subfields may help elucidate the regions involved in specific memory functions.

Extensive studies have reported that the volumes of hippocampal subfields non-linearly decreased with aging. Moreover, hippocampal changes in different subfields are inconsistent (Malykhin et al., 2017; Zheng et al., 2018). Daugherty et al. (2016) observed that the volumes of CA1-2 and CA3-DG significantly decreased with increasing age. However, Malykhin et al. (2017) found no significant difference in CA1-3, but did report marked changes in the subiculum and DG. Others reported volume atrophy in the hippocampus of patients with AD (Wisse et al., 2014; Kälin et al., 2017; Platero et al., 2018). For instance, Wisse et al. (2014) found that AD patients exhibited smaller volumes in the subiculum, CA1, CA3 and DG/CA4. DLB has pathologic overlap with AD (Mckeith et al., 2005). The results of a study of a mouse model expressing mutant β-synuclein (linked to DLB) suggested that the pseudo-immaturity of DG granule cells may be a shared endophenotype in patients with neurodegenerative disorders (Hagihara et al., 2018). Several groups have found that hippocampal atrophy is less severe in DLB than AD (Firbank et al., 2010; Elder et al., 2017; Pang et al., 2018). For instance, Firbank et al. (2010) found that the CA1 and subiculum exhibited less atrophy in DLB patients compared to AD patients. These observations underscore the importance of identifying biomarkers to distinguish between normal and pathological aging.

Researchers have investigated the relationships between hippocampal subfields and memory in case control studies, but few have focused on age-related memory decline (Mueller et al., 2012; Comper et al., 2017). Thus, the association between age-related memory decline and hippocampal subfield volumes in normal humans remains unclear. In this study, an automatic method in Freesurfer software was used to segment the hippocampus into 13 subfields in each hemisphere on 275 normal adults (age 20–89) imaged at the same scanning center. The main purpose was to investigate volume changes in hippocampal subfields across the adult lifespan and examine their associations with memory recall decline based on immediate and delayed recall scores.

## Materials and Methods

### Participants

T1-weighted MRI data of 315 healthy adults, aged 20–89 years, were selected from the DLBS^1^. Subjects with incomplete cognitive information were excluded. Finally, total 275 subjects (54.85 ± 20.61 years; 174 females, 101 males) were included in the present study. The population were classified into 4 groups: Young group, 20–35 years; Middle-early, 36–50 years; Middle-late, 51–65 years; Old group, 66–89 years. Participants were recruited through flyers and media advertisements. Participants were right-handed and native English speakers with no history of neurological disease. The participants were well-educated and with high scores on the Mini-Mental State Examination (MMSE > 26). Immediate and Delayed recall abilities were measured with Hopkins Verbal Learning Scoring (Brandt, 1991), and a 20-min delay was used for Delayed recall score measurement. This experiment was approved by the Institutional Review Board of the University of Texas Southwestern Medical Center and the University of Texas at Dallas. All participants provided written informed consent.

### sMRI Data Acquisition

All participants underwent T1-weighted imaging on a Philips Achieva 3T scanner (Amsterdam, Netherlands). The parameters were as follows: slice thickness = 1 mm, repetition time = 8.135 ms, echo time = 3.7 ms, matrix = 256 × 256, field of view = 204 × 256. The direction was anterior-posterior.

### Imaging Processing

**FIGURE 1:**
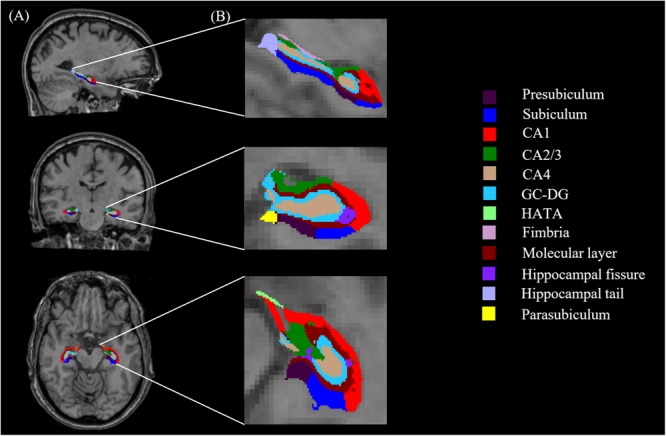
Hippocampal segmentation. **(A)** T_1_ images of hippocampal subfields in the sagittal, coronal, and axial planes. **(B)** Magnified views.

All T1-weighted images were processed using publicly available Freesurfer software^2^. We conducted the main recon stream (“recon-all”) in Freesurfer 6.0 for volumetric segmentation, including motion correction, skull stripped, intensity normalization, automated Talairach transformation, gray/white matter tessellation, and topology correction (Fischl et al., 2001; Segonne et al., 2007). Subcortical structures were segmented with a non-linear warping atlas (Macdonald, 1997; Fischl et al., 2016), and total hippocampal volumes were obtained. Subsequently, a probabilistic atlas and a modified version of Van Leemput’s algorithm were applied to segment the hippocampus (Van Leemput et al., 2009; Iglesias et al., 2015; Saygin et al., 2017) into 13 subfields in each hemisphere: the CA1, CA2/3, CA4, molecular layer, alveus, GC-DG, HATA, subiculum, presubiculum, parasubiculum, fimbria, hippocampal tail and fissure, as shown in **Figure 1**. The CA2 and CA3 were combined because of unclear contrast, and the alveus volume was removed due to the thin shape and unreliable segmentation (Iglesias et al., 2015).

### Statistical Analysis

Statistical analysis was performed using IBM SPSS software (version 22.0, Armonk, NY, United States) and MATLAB (MathWorks Inc., Natick, MA, United States). Chi-square tests were used to evaluate the differences in gender distribution among groups. One-way ANOVA was performed to assess group differences in education years, while ANCOVA was performed for the differences in MMSE scores, Immediate and Delayed recall scores among the 4 groups, with gender and education years as covariates. For hippocampal subfield volume differences, we considered gender, education years and eTIV as covariates for ANCOVA was performed. Regarding the indexes with significant differences among the 4 groups, multiple comparison between groups was performed using the Bonferroni method. Spearman correlation analyses were applied in MATLAB using a home-written program to verify the correlations between memory recall scores and hippocampal subfields, and correlation coefficients (R) were calculated. Gender, education years, and eTIV were also regarded as covariates for the correlations between memory recall scores and hippocampal subfields. Spearman correlation results were corrected by FDR correction in MATLAB. The significance level of all results was set at *P* < 0.05.

## Results

### Differences in Demographics and Recall Scores

The demographic characteristics and Immediate recall and Delayed recall scores for 275 subjects are shown in **Table 1** as means and standard deviations. MMSE scores, although still within the normal range, significantly declined with age (*P* < 0.001). There were significant differences in MMSE scores (*P* < 0.001), Immediate recall scores (*P* < 0.001) and Delayed recall scores (*P* = 0.003). Furthermore, the pairwise comparisons demonstrated apparent declines in MMSE and Immediate recall scores in the Old group (*P* < 0.05) compared with other groups, as well as lower Delayed recall scores (*P* < 0.05) compared with the Young and Middle-late groups, as shown in **Supplementary Table 1**. No significant difference was observed for gender distribution (*P* = 0.462) or education years (*P* = 0.338) among the 4 groups.

**Table 1:** Demographic information of the 4 groups.

	Young (*N* = 71)	Middle-early (*N* = 49)	Middle-late (*N* = 54)	Old (*N* = 101)	χ^2&^/*F*^a^	*P*^a^	*F*^b^	*P*^b^
Age	27.85 ± 4.82	43.61 ± 4.67	58.57 ± 4.60	77.16 ± 6.67	n.d.	n.d.	n.d.	n.d.
Gender(F/M)	45/26	29/20	39/15	61/40	2.574^&^	0.462	n.d.	n.d.
Education	16.38 ± 2.29	16.07 ± 2.16	16.70 ± 1.95	16.06 ± 2.46	1.128	0.338	n.d.	n.d.
MMSE	28.73 ± 1.17	28.69 ± 1.18	28.50 ± 1.10	27.63 ± 1.25	16.046	<0.001^∗∗^	15.499	<0.001^∗∗^
Immediate recall	7.93 ± 1.80	7.35 ± 2.02	7.39 ± 1.97	6.47 ± 1.60	9.753	<0.001^∗∗^	9.340	<0.001^∗∗^
Delayed recall	6.21 ± 2.70	5.47 ± 3.00	6.02 ± 2.45	4.72 ± 2.34	5.591	0.001^∗^	4.818	0.003^∗^

*Data expressed as mean ± SD. N, the number of participants. MMSE, Mini-Mental State Exam; n.d., not done.^∗^*P* < 0.05, ^∗∗^*P* < 0.001. ^&^Chi-square test. ^*a*^ANOVA test, no covariates. ^*b*^ANCOVA test, controlling for gender and education years.*

### Age Effects on Hippocampal Subfield Volume

**FIGURE 2:**
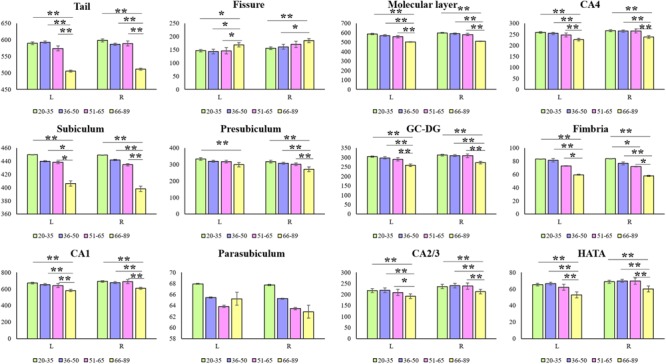
Mean volumes of hippocampal subfields in the Young, Middle-early, Middle-late, and Old groups. L, left hemisphere; R, right hemisphere; The *Y*-axis indicates the mean volume of hippocampal subfields in each group. ^∗^*P* < 0.05, ^∗∗^*P* < 0.001; 95% confidence intervals.

Hippocampal subfield volumes change with aging. **Table 2** summarizes the statistical analysis for the volume of the hippocampal subfields. Besides no significant difference in the left parasubiculum (*P* = 0.269) and a slight difference in the right parasubiculum (*P* = 0.022), other subfields were significantly different among the 4 groups (*P* < 0.001). **Supplementary Table 2** lists the statistical results of pairwise comparisons in subfields with significant differences among the 4 groups. Histograms in **Figure 2** demonstrate pairwise comparisons on hippocampal subfield volumes. Most were significantly decreased in the Old group compared with the other 3 groups, including the bilateral hippocampal tail, subiculum, CA1, molecular layer, GC-DG, CA2/3, CA4, fimbria, HATA, and right presubiculum (*P* < 0.05). The left presubiculum volume was only significantly different between the Old and Young groups (*P* < 0.001). A significant decline of right fimbria volume was also observed in the Middle-late group relative to the Young group (*P* = 0.015). Interestingly, the hippocampal fissure volume was increased in the Old group relative to other groups for the left hemisphere (*P* < 0.05), as well as relative to the Young and Middle-early groups for the right (*P* < 0.05). In addition, although there were slight differences among the 4 groups in right parasubiculum, no significance was found between groups (*P* > 0.05).

**Table 2:** Statistical analysis of hippocampal subfields volumes in all study subjects.

	L				R			
	*F*^a^	*P*^a^	*F*^b^	*P*^b^	*F*^a^	*P*^a^	*F*^b^	*P*^b^
Whole hippocampus	25.877	<0.001^∗∗^	40.846	<0.001^∗∗^	26.959	<0.001^∗∗^	41.898	<0.001^∗∗^
Hippocampal_tail	24.855	<0.001^∗∗^	27.915	<0.001^∗∗^	23.659	<0.001^∗∗^	30.256	<0.001^∗∗^
Subiculum	8.825	<0.001^∗∗^	14.900	<0.001^∗∗^	13.373	<0.001^∗∗^	20.950	<0.001^∗∗^
CA1	18.727	<0.001^∗∗^	29.399	<0.001^∗∗^	17.917	<0.001^∗∗^	27.387	<0.001^∗∗^
Fissure	8.215	<0.001^∗∗^	6.645	<0.001^∗∗^	6.647	<0.001^∗∗^	6.145	<0.001^∗∗^
Presubiculum	6.716	<0.001^∗∗^	10.139	<0.001^∗∗^	17.953	<0.001^∗∗^	22.937	<0.001^∗∗^
Parasubiculum	1.115	0.344	1.317	0.269	2.525	0.058	3.252	0.022^∗^
Molecular_layer	25.938	<0.001^∗∗^	40.162	<0.001^∗∗^	29.673	<0.001^∗∗^	43.105	<0.001^∗∗^
GC-DG	30.065	<0.001^∗∗^	46.173	<0.001^∗∗^	22.503	<0.001^∗∗^	35.153	<0.001^∗∗^
CA2/3	12.748	<0.001^∗∗^	20.989	<0.001^∗∗^	12.259	<0.001^∗∗^	20.493	<0.001^∗∗^
CA4	21.250	<0.001^∗∗^	33.208	<0.001^∗∗^	15.714	<0.001^∗∗^	25.648	<0.001^∗∗^
Fimbria	19.177	<0.001^∗∗^	23.116	<0.001^∗∗^	21.427	<0.001^∗∗^	24.581	<0.001^∗∗^
HATA	28.240	<0.001^∗∗^	36.114	<0.001^∗∗^	13.406	<0.001^∗∗^	19.637	<0.001^∗∗^

*L, left hemisphere; R, right hemisphere. ^∗^*P* < 0.05, ^∗∗^*P* < 0.001. ^*a*^ANOVA test, no covariates. ^*b*^ANCOVA test, controlling for gender, education years and eTIV.*

### Associations Between Memory Recall Scores and Hippocampal Subfields

**Table 3** lists the Spearman correlation analysis results. There were significant correlations between bilateral whole hippocampal volume and Immediate and Delayed recall scores (*P* < 0.05). Furthermore, the results showed that regarding Immediate recall scores, there were positive correlations with the bilateral molecular layer, GC-DG, CA1, CA2/3 and CA4, left fimbria and HATA (*P* < 0.05). In addition, a negative association was observed for the right fissure (*R* = -0.1411, *P* = 0.0358). Delayed recall scores were strongly and positively associated with the bilateral GC-DG, molecular layer, CA2/3 and CA4, left tail, presubiculum, subiculum, CA1, fimbria and HATA (*P* < 0.05). In addition, we found that 10 subfields on the left were related to Delayed recall scores, compared to 4 on the right.

**Table 3:** Correlation analysis between hippocampal subfield volumes and Immediate recall and Delayed recall scores.

	Immediate recall		Delayed recall	
	*R*	*P*	*R*	*P*
Left_whole	0.1797	0.0104*	0.1925	0.0104*
Left_tail	0.1198	0.0663	0.1585	0.0210*
Left_subiculum	0.1259	0.058	0.1648	0.0170*
Left_CA1	0.1803	0.0104*	0.1737	0.0113*
Left_fissure	–0.0576	0.3838	–0.0594	0.3769
Left_presubiculum	0.1127	0.0825	0.1353	0.0432*
Left_parasubiculum	0.0140	0.8334	0.0246	0.7124
Left_molecular layer	0.1899	0.0104*	0.2024	0.0104*
Left_GC-DG	0.1804	0.0104*	0.1960	0.0104*
Left_CA2/3	0.1316	0.0488*	0.1566	0.0222*
Left_CA4	0.1543	0.0237*	0.1758	0.0107*
Left_fimbria	0.1800	0.0104*	0.1356	0.0432*
Left_HATA	0.1992	0.0104*	0.1811	0.0104*
Right_whole	0.1634	0.0174*	0.1469	0.0308*
Right_tail	0.0951	0.1431	0.1253	0.0580
Right_subiculum	0.0856	0.1895	0.0952	0.1431
Right_CA1	0.1415	0.0358*	0.1029	0.1151
Right_fissure	–0.1411	0.0358*	–0.0561	0.3838
Right_presubiculum	0.1290	0.0528	0.1233	0.0595
Right_parasubiculum	–0.0339	0.6106	0.0051	0.9324
Right_molecular layer	0.1904	0.0104*	0.1514	0.0260*
Right_GC-DG	0.1968	0.0104*	0.1788	0.0104*
Right_CA2/3	0.1762	0.0107*	0.1436	0.0344*
Right_CA4	0.1862	0.0104*	0.1809	0.0104*
Right_fimbria	0.1239	0.0595	0.0560	0.3838
Right_HATA	0.1188	0.0673	0.0647	0.3362

*R, correlation coefficient. ^∗^*P* < 0.05.*

## Discussion

In this study, we measured hippocampal subfield volume changes and their correlations with Immediate recall and Delayed recall scores across the adult lifespan. Nearly all volumes showed significant declines in the Old group except the left parasubiculum, while there was a significant increase in the fissure volume. Several hippocampal subfields were significantly related to Immediate and Delayed recall scores. Moreover, we also observed a stronger relationship between delayed recall scores and left hippocampus volumes.

### Age Effects on Hippocampal Subfield Volumes

Differences in segmentation methods impact the number and volume of hippocampal subfields reported. A popular segmentation method yields the CA1-4, DG, and subiculum (Perrotin et al., 2015; Riggins et al., 2018), as well as the presubiculum in some studies (de Flores et al., 2014; Carlesimo et al., 2015). We performed the more detailed segmentation proposed by Iglesias et al. (2015) that includes the CA1, CA2/3, CA4, molecular layer, alveus, GC-DG, HATA, subiculum, presubiculum, parasubiculum, fimbria, tail, and fissure.

Previous studies reported that the effects of age impact several specific subfields rather than the entire hippocampus (de Flores et al., 2014; Wisse et al., 2014; Malykhin et al., 2017). de Flores et al. (2014) manually segmented the hippocampus into the CA1, subiculum, and other regions. They observed that CA1 volume non-linearly decreased with aging and dropped at about 50 years, and subiculum volume linearly decreased with aging; no other subfields exhibited volume decreases. Malykhin et al. (2017) subsequently described negative correlations between the total subiculum and DG volumes with aging, whereas there was no significance for the total CA1-3. We observed significant differences in volumes of the CA1, CA2/3, CA4, GC-DG, and subiculum. One possible reason for the differences is that de Flores et al. (2014) combined the CA2/3/4 and DG into a single region of interest (ROI) called “others,” while Malykhin et al. (2017) combined CA1, 2 and 3 into a single ROI called “CA1-3.” Different subfield combinations may obscure age-related changes of single subfields. Additionally, animal studies revealed that fimbria lesions may impair object discrimination (Wible et al., 1992; Antoniadis and Mcdonald, 2006). Extrapolating these animal studies to humans, the reduction of fimbria volume in this study might be considered as an early biomarker for visual dysfunction.

Using the same segmentation method, significant non-linear age-related declines in CA2/3, CA4 and GC-DG volumes, and a significant linear increase in fissure volume were recently reported (Zheng et al., 2018). They found no significant difference in parasubiculum volume. These are similar to our results, but Zheng and colleagues found no significant volume differences in the hippocampal tail, presubiculum, subiculum, CA1, molecular layer, fimbria, or HATA. However, according to their raw plots and change trends, a non-linear decline trend was evident. The main reason underlying the discrepant results might be the dataset; they included 54 subjects, while we assessed 275.

Decreased hippocampal subfield volumes have been widely reported in dementia disorders such as AD and DLB (Delli Pizzi et al., 2016; Mak et al., 2016, 2017). One group found that the volumes of the CA1, CA2-3, CA4, DG, and total subiculum (subiculum, presubiculum, and parasubiculum) are decreased in AD (Mak et al., 2017). In the present study, the volumes of all subfields except the parasubiculum were decreased in the Old group, which is similar to observations in subjects with dementia disorders. This suggests that these subfields may not be useful biomarkers to investigate normal aging and dementia. According to our results, only the parasubiculum did not show significant atrophy in the Old group, indicating its use as a dementia biomarker. This is supported by prior studies implicating the parasubiculum involved in dementia, as well as its important role in the medial temporal memory system (Caballerobleda and Witter, 1993; Glasgow and Chapman, 2007; Ding, 2013). In an immunohistochemistry study of an AD mouse model, Weidensteiner and his colleagues identified plaque depositions in the subiculum/parasubiculum (Weidensteiner et al., 2009). Earlier research reports described severe pathology in the parasubiculum of subjects with Creutzfeldt-Jakob disease (Guentchev et al., 1997; Kaneko et al., 1999). In addition, Fukutani et al. (1995) reported that compared to healthy subjects, AD patients had fewer unaffected neurons and more intra and extracellular neurofibrillary tangles in the parasubiculum, entorhinal cortex, prosubiculum and CA1. Other studies reported similar results noting severely affected neurons in the parasubiculum of AD brains (Chanpalay et al., 1986; Yamaguchi et al., 1988; Kalus et al., 1989; Fukutani et al., 2000). Iglesias et al. (2016) described parasubiculum atrophy in AD patients. Regarding AD progression, Dong et al. (2018) reported that parasubiculum volume is significantly correlated with neuropsychological test scores in patients with amnestic mild cognitive impairment. Based on these findings, we hypothesize that the parasubiculum may be a potential biomarker for dementia disorders. Further research is necessary to replicate these findings in other samples and advance our understanding.

### Associations Between Memory and Hippocampal Subfields

Hippocampal subfield volumes are closely correlated with memory ability (Daugherty et al., 2017). The strong association between the CA1 and memory performance was previously reported (Adamowicz et al., 2017). Furthermore, neuronal density in the CA1 was significantly associated with preoperative Immediate and Delayed recall scores in patients undergoing temporal lobectomy (Baxendale et al., 1998). Another study reported that the CA1 is involved in t Immediate and Delayed recall in patients with left hippocampal sclerosis (Comper et al., 2017). Notably, we obtained similar results in healthy subjects. Other studies reached different conclusions. Animal literature supports a role of the CA1 in intermediate and long-term memory but not short-term memory (Remondes and Schuman, 2004; Vago et al., 2007). One group compared healthy and cognitively impaired elderly subjects and concluded that the CA1 is associated with Delayed recall Discriminability but not Immediate or Short Free Recall Discriminability, while, the CA3 and DG are associated with Immediate and Short Free Recall Discriminability (Mueller et al., 2011). In our study, the CA1, CA2/3 and GC-DG were related to both Immediate and Delayed recall scores. There are two possible explanations for this discrepancy. Firstly, their study only included 50 subjects. Secondly, some of their subjects with cognitive impairment might have been affected by AD, which could affect structure-memory associations. In accordance with our results, Finke et al. (2016) found that the decreased Delayed recall scores were accompanied by atrophy of the CA2/3 and CA4/DG in encephalitis patients with leucine-rich, glioma-inactivated 1 antibodies.

Another study suggested that conversion of patients with PD from no cognitive impairment to mild cognitive impairment was related to the baseline volumes of the GC-DG, left parasubiculum, left HATA, and right CA4. The authors postulated that parasubiculum and HATA atrophy might affect the integrity of hippocampal-amygdala network that underlies information processed (Foo et al., 2017). Their results indicate that these subfields might be critical to cognition. Additionally, preliminary animal studies reported that the parasubiculum may be more involved in online spatial information processing rather than long-term information storage (Kesner and Giles, 1998; Liu et al., 2004; Tang et al., 2016). However, we did not find significant correlations between recall scores and parasubiculum volume. This discrepancy may be due to variable ways of measuring cognition or subject differences.

Lim et al. (2013) found that lower volumes of the subiculum and presubiculum predicted poorer Delayed verbal recall ability in PD patients. Subsequently, Stav and colleagues reached a similar conclusion in AD patients (Stav et al., 2016). These studies indicate that the subiculum and presubiculum might be related to Delayed verbal recall ability, which is consistent with our results, although we only observed a slight association for the presubiculum.

The molecular layer including neurons of the subiculum and CA fields lies above the subiculum and underneath the fissure. Few studies have reported correlations between molecular layer volume and Immediate and Delayed recall abilities. Cantero et al. (2016) found that lower volumes of the DG and molecular layer were associated with Delayed memory. A recent study concluded that molecular layer volume was positively associated with general cognition from childhood to adulthood (Tamnes et al., 2018). Therefore, we considered that the current study might reach more accurate conclusions about the impacts of molecular layer volume on memory function. One group found that the number of synapses in molecular layer was highly correlated with Delayed recall and Delayed recognition abilities, but there was no association with Immediate recall ability (Scheff et al., 2006). In our study, we observed that molecular layer volume was correlated with both Immediate and Delayed recall abilities. We plan to perform molecular biology experiments to explore the relationship between this brain region and memory.

We found that Immediate and Delayed recall scores were associated with the volume of left fimbria, a white matter structure that extends from the alveus and eventually forms the fornix. Consistent with our results, rats with fimbria-fornix lesions exhibited short-term memory impairment (Winters and Dunnett, 2004; Addy et al., 2005). Moreover, Tomaiuolo et al. (2004) observed that Immediate and Delayed recall scores were both associated with fornix volume.

Our results should be considered in the context of several limitations. First, there is a gender distribution imbalance, with data from 101 males and 174 females. In addition, our subjects were aged 20–89 years and lack of development children; and this should be addressed in further studies. Second, vascular risk increases with aging, and the effect of blood pressure on hippocampus volume is not negligible (Shing et al., 2011). All data included in this study were acquired from DLBS dataset, so we were unable to obtain more clinical information, which may have influenced our results. Third, this was a cross-sectional study; longitudinal assessments will be necessary to confirm our findings. Moreover, there are some limitations regarding the segmentation method. Since the atlas was developing using data from elderly subjects, there may be slight hippocampal atrophy. Finally, although the MRI scans were ultra-high resolution MRI, there were some unclear boundaries between subfields, such as CA fields or the interface between CA4/GC-DG.

## Conclusion

We explored hippocampal subfield volumes alterations in subjects of different ages and correlated these changes with Immediate recall and Delayed recall scores. Multiple hippocampal subfields were smaller in the Old group, in addition to parasubiculum, which is reportedly atrophied in some dementia disorders. We therefore speculate that a significant decline in parasubiculum volume maybe a potential biomarker for dementia disorders, but further investigation is needed to support this hypothesis. Taken together, our results indicate that subfield volume changes are related to age-related memory decline and might be regarded as biomarkers in postponing memory decline across the adult lifespan.

## Data Availability Statement

The datasets for this study can be found in the Dallas Lifespan Brain Study (DLBS) [http://fcon_1000.projects.nitrc.org/indi/retro/dlbs.html].

## Ethics Statement

The data included in this study was obtained from the Dallas Lifespan Brain Study (DLBS), which is available on the International Neuroimaging Data-sharing Initiative (INDI). This study was carried out in accordance with the recommendations of ’UT Southwestern Institutional Review Board’ with written informed consent from all subjects. All subjects gave written informed consent in accordance with the Declaration of Helsinki. The protocol was approved by the UT Southwestern Institutional Review Board.

## Author Contributions

DC and TY designed the study. FZ performed the imaging processing and wrote the manuscript. ZmL and DZ organized and filtered the data. LZ, SZ, and YZ analyzed the data. XL and DC contributed to the statistical analysis. DC, CL, LS, ZpL, KH, WL, and JQ reviewed and edited the manuscript. All the authors read and approved the submitted manuscript.

## Conflict of Interest Statement

The authors declare that the research was conducted in the absence of any commercial or financial relationships that could be construed as a potential conflict of interest.

## Footnotes

1 http://fcon_1000.projects.nitrc.org/indi/indiPRIME.html

2 http://surfer.nmr.mgh.harvard.edu

## Abbreviations

AD: Alzheimer’s disease
ANCOVA: analysis of covariance
ANOVA: analysis of variance
CA: cornus ammonis
DG: dentate gyrus
DLB: dementia with Lewy bodies
DLBS: Dallas Lifespan Brain Study
eTIV: estimated total intracranial volume
FDR: false discovery rate
GC-DG: granule cell layer of the dentate gyrus
HATA: hippocampal amygdala transition area
MMSE: Mini-Mental State Examination
MRI: magnetic resonance imaging
PD: Parkinson’s disease
