# Supplementary material for: The Volume of Hippocampal Subfields in Relation to Decline of Memory Recall Across the Adult Lifespan
Source: Front Aging Neurosci. 2018 Oct 10;10:320. doi: 10.3389/fnagi.2018.00320 (PMC6191512; doi:10.3389/fnagi.2018.00320)
Supplement: Supplementary file 1 [file Table_1.docx]

Supplementary Material

The volume of hippocampal subfields in relation to decline of memory recall across the adult lifespan

**Fenglian Zheng**^1,2,3^**^#^, Dong Cui**^4,3^**^#^, Li Zhang**^1,2,3^**, Shitong Zhang**^5,1,2,3^**, Yue Zhao**^5,1,2,3^**, Xiaojing Liu**^1,2,3^**, Chunhua Liu**^6^**, Zhengmei Li**^1,2,3^**, Dongsheng Zhang**^1,2,3^**, Liting Shi**^1,2,3^**, Zhipeng Liu**^4^**, Kun Hou**^1,2,3^**, Wen Lu**^1,2,3^**, Tao Yin**^4^**^*^, Jianfeng Qiu**^1,2,3^**^*^**

^1^Medical engineering and technology Research Center, Taishan Medical University, Taian, China

^2^Imaging-X Joint Laboratory, Taian, China

^3^College of Radiology, Taishan Medical University, Taian, China

^4^Institute of Biomedical Engineering, Chinese Academy of Medical Sciences and Peking Union Medical College, Tianjin, China

^5^College of Mechanical and Electronic Engineering, Shandong University of Science and Technology, Qingdao, China

^6^School of Basic Medical Sciences, Taishan Medical University, Taian, China

^#^ These authors contributed equally to this work.

*** Correspondence:** Jianfeng Qiu: [jfqiu100@gmail.com](mailto:jfqiu100@gmail.com); Tao Yin: [bme500@163.com](mailto:bme500@163.com)

# Supplementary Tables

**Supplementary Table 1.** The pairwise comparisons of MMSE, Immediate recall and Delayed recall scores between Young group, Middle-early group, Middle-late group and Old group.

|  | Young v Middle-early | Young v Middle-late | Young v Old | Middle-early v Middle-late | Middle-early v Old | Middle-late v Old |
| --- | --- | --- | --- | --- | --- | --- |
| MMSE | 1.000 | 1.000 | <0.001^**^ | 1.000 | <0.001^**^ | <0.001^**^ |
| Immediate recall | 0.499 | 0.588 | <0.001^**^ | 1.000 | 0.032^*^ | 0.016^*^ |
| Delayed recall | 0.735 | 1.000 | 0.001^*^ | 1.000 | 0.584 | 0.019^*^ |

Note: The expressed data is P value of the pairwise comparisons between groups, using Bonferroni method. ^*^ P < 0.05, ^**^ P < 0.001.
