# Supplementary material for: The Volume of Hippocampal Subfields in Relation to Decline of Memory Recall Across the Adult Lifespan
Source: Front Aging Neurosci. 2018 Oct 10;10:320. doi: 10.3389/fnagi.2018.00320 (PMC6191512; doi:10.3389/fnagi.2018.00320)
Supplement: Supplementary file 2 [file Table_2.docx]

Supplementary Material

The volume of hippocampal subfields in relation to decline of memory recall across the adult lifespan

**Fenglian Zheng**^1,2,3^**^#^, Dong Cui**^4,3^**^#^, Li Zhang**^1,2,3^**, Shitong Zhang**^5,1,2,3^**, Yue Zhao**^5,1,2,3^**, Xiaojing Liu**^1,2,3^**, Chunhua Liu**^6^**, Zhengmei Li**^1,2,3^**, Dongsheng Zhang**^1,2,3^**, Liting Shi**^1,2,3^**, Zhipeng Liu**^4^**, Kun Hou**^1,2,3^**, Wen Lu**^1,2,3^**, Tao Yin**^4^**^*^, Jianfeng Qiu**^1,2,3^**^*^**

^1^Medical engineering and technology Research Center, Taishan Medical University, Taian, China

^2^Imaging-X Joint Laboratory, Taian, China

^3^College of Radiology, Taishan Medical University, Taian, China

^4^Institute of Biomedical Engineering, Chinese Academy of Medical Sciences and Peking Union Medical College, Tianjin, China

^5^College of Mechanical and Electronic Engineering, Shandong University of Science and Technology, Qingdao, China

^6^School of Basic Medical Sciences, Taishan Medical University, Taian, China

^#^ These authors contributed equally to this work.

*** Correspondence:** Jianfeng Qiu: [jfqiu100@gmail.com](mailto:jfqiu100@gmail.com); Tao Yin: [bme500@163.com](mailto:bme500@163.com)

# Supplementary Tables

**Supplementary Table 2.** The pairwise comparisons of hippocampal subfields volume between Young group, Middle-early group, Middle-late group and Old group.

|  | Young v Middle-early | Young v Middle-late | Young v Old | Middle-early v Middle-late | Middle-early v Old | Middle-late v Old |
| --- | --- | --- | --- | --- | --- | --- |
| Left_whole | 1.000 | 0.169 | <0.001^**^ | 1.000 | <0.001^**^ | <0.001^**^ |
| Left_tail | 1.000 | 1.000 | <0.001^**^ | 1.000 | <0.001^**^ | <0.001^**^ |
| Left_subiculum | 1.000 | 1.000 | <0.001^**^ | 1.000 | 0.007^*^ | 0.009^*^ |
| Left_CA1 | 1.000 | 0.199 | <0.001^**^ | 1.000 | <0.001^**^ | <0.001^**^ |
| Left_fissure | 1.000 | 1.000 | 0.001^*^ | 1.000 | 0.001^*^ | 0.003^*^ |
| Left_presubiculum | 1.000 | 0.652 | <0.001^**^ | 1.000 | 0.092 | 0.137 |
| Left_molecular layer | 1.000 | 0.120 | <0.001^**^ | 1.000 | <0.001^**^ | <0.001^**^ |
| Left_GC-DG | 1.000 | 0.133 | <0.001^**^ | 1.000 | <0.001^**^ | <0.001^**^ |
| Left_CA2/3 | 1.000 | 0.778 | <0.001^**^ | 0.620 | <0.001^**^ | 0.010^*^ |
| Left_CA4 | 1.000 | 0.221 | <0.001^**^ | 1.000 | <0.001^**^ | <0.001^**^ |
| Left_fimbria | 1.000 | 0.064 | <0.001^**^ | 0.315 | <0.001^**^ | 0.003^*^ |
| Left_HATA | 1.000 | 0.595 | <0.001^**^ | 0.211 | <0.001^**^ | <0.001^**^ |
| Right _whole | 1.000 | 1.000 | <0.001^**^ | 1.000 | <0.001^**^ | <0.001^**^ |
| Right_tail | 1.000 | 1.000 | <0.001^**^ | 1.000 | <0.001^**^ | <0.001^**^ |
| Right_subiculum | 1.000 | 0.978 | <0.001^**^ | 1.000 | <0.001^**^ | 0.001^*^ |
| Right_CA1 | 1.000 | 1.000 | <0.001^**^ | 1.000 | <0.001^**^ | <0.001^**^ |
| Right_ fissure | 1.000 | 0.428 | <0.001^**^ | 1.000 | 0.015^*^ | 0.369 |
| Right_presubiculum | 1.000 | 0.338 | <0.001^**^ | 1.000 | <0.001^**^ | <0.001^**^ |
| Right_parasubiculum | 1.000 | 0.293 | 0.056 | 1.000 | 1.000 | 1.000 |
| Right_molecular layer | 1.000 | 0.968 | <0.001^**^ | 1.000 | <0.001^**^ | <0.001^**^ |
| Right_GC-DG | 1.000 | 1.000 | <0.001^**^ | 1.000 | <0.001^**^ | <0.001^**^ |
| Right_CA2/3 | 1.000 | 1.000 | <0.001^**^ | 1.000 | <0.001^**^ | <0.001^**^ |
| Right_CA4 | 1.000 | 1.000 | <0.001^**^ | 1.000 | <0.001^**^ | <0.001^**^ |
| Right_fimbria | 0.478 | 0.015^*^ | <0.001^**^ | 1.000 | <0.001^**^ | 0.001^*^ |
| Right_HATA | 1.000 | 1.000 | <0.001^**^ | 1.000 | <0.001^**^ | <0.001^**^ |

Note: The expressed data is P value of the pairwise comparisons between groups, using Bonferroni method. ^*^ P < 0.05, ^**^ P < 0.001.
